# Report from the Scientific Poster Session at the 15th Annual Cardiometabolic Health Congress Live Online, USA, 21–24 October 2020

**DOI:** 10.3390/medsci8040050

**Published:** 2020-12-14

**Authors:** Organizing Committee Cardiometabolic Health Congress Annual 2020

**Affiliations:** Tarsus Cardio dba Cardiometabolic Health Congress (CMHC), Boca Raton, FL 33431, USA; info@cardiometabolichealth.org; Tel.: +1-888-997-0112

## 1. Preface

The 15th Annual CMHC Live Online concluded on Saturday, October 24th. For the first time in its history, the event was delivered entirely online to practitioners across the nation. Through an innovative and user-friendly virtual platform, participants were able to interact with faculty members, participate in audience polls, and more, all from the setting of their own choosing. Chaired by an esteemed panel of expert faculty members Robert H. Eckel, MD; Christie M. Ballantyne, MD; George L. Bakris, MD; Anne L. Peters, MD; Deepak L. Bhatt, MD, MPH; Keith C. Ferdinand, MD; Clyde W. Yancy, MD, MSc, MACP; and Pamela B. Morris, MD, the conference provided actionable insights on late-breaking research, advanced clinical approaches to cardiometabolic challenges, and practical strategies to tackle inequity in healthcare. 

Developed and led by the foremost clinicians, researchers, and industry thought leaders, the 15th Annual Cardiometabolic Health Congress delivered top-tier clinical perspectives from a myriad of specialties. Designed in direct response to the real-world clinical needs of active cardiometabolic practitioners and patients of all backgrounds, this cutting-edge event disseminated the latest science and clinically equitable strategies in cardiometabolic medicine available. 

In addition to offering cutting-edge and comprehensive education, the 2020 CMHC hosted its fourth annual Scientific Poster Session, where investigators from around the world brought the latest data from current research and clinical findings to share with attendees.

## 2. Keynote Poster Abstracts

### 2.1. Quantifying the Expected versus the Observed Return on Investment in Healthcare Policy and Programming in Populations over 65 Years—Case Study in Diabetes Mellitus

MeierGenevieveSchool of Medicine, Cardiff University, Cardiff, UK

**Purpose:** Analyze the real-world impact of clinical guidelines on outcomes, using a before–after study design limited to diabetes management in patients older than 65 years.

**Method**: Systematically identify all the policies and guidelines that have a direct impact on those older than 65 years with type-2 diabetes in terms of quality of care, the date and rationale of their introduction. A retrospective before and after analysis was performed using the Clinical Practice Research Datalink (CPRD) database pre- and post-introduction of each policy/guideline since 2000 in a stepwise manner. The outcomes of interest were hospitalizations, cases of large-vessel disease, cases of type-2 diabetes micro-vascular complications, cases of cancer and mortality. 

**Results**: Guideline 2002 had no impact on large-vessel disease events or cancers, however, it did have a negative impact on hospitalizations, (HR = 1.34, *p* = 0.025), as well as on mortality (HR = 4.14, *p* < 0.001). Guideline 2008 had no impact on hospitalizations, however it did have a positive impact on large-vessel disease (HR = 0.85, *p* = 0.01), cancers (HR = 0.77, *p* = 0.009) and mortality (HR = 0.63, *p* < 0.001). Nonetheless, rates did not return to pre-2002 levels for mortality. Guideline 2012 had no meaningful impact on hospitalizations, cases of large-vessel disease, cases of cancer or mortality. All guidelines were associated with an increase in microvascular complications.

**Conclusions**: Guidelines for type-2 diabetes since 2000 have not been successful overall in improving patient care and other important outcomes such as hospitalizations, cases of large-vessel disease, cases of cancer and mortality. Before and after real-world studies could assist in the review of guidelines and in their ongoing development.

### 2.2. Obesity Perception and Primary Care Interventions in Patients Who Were Referred to the Internal Medicine Outpatient Clinic of the Hospital Universidad del Norte (HUN)

RuizEder Augusto Hernandez^1^VelezJuan Gabriel Acosta^2^ParejaDaniela Lucia Varela^3^1Obesity and Metabolic Syndrome Clinic, Hospital Universidad del Norte, Barranquilla, Atlántico, Colombia2Health Sciences Education, McMaster University, Hamilton, ON, Canada3Obesity and Metabolic Syndrome Clinic, Hospital Universidad del Norte, Barranquilla, Atlántico, Colombia

**Purpose**: To identify patient's perception about their weight, as well as interventions carried out in primary care to achieve a healthy weight and lifestyle modification.

**Method**: This was a cross-sectional descriptive observational study. Overweight and obese patients referred to the Internal Medicine outpatient clinic at HUN were included. The patients were asked to fill a questionnaire inquiring about self-perception of weight and their opinion regarding interventions such as nutritional assessment, exercise prescription and other weight loss strategies.

**Results**: One hundred patients were included; 66% were women, 46% were obese, only 14% had a prior diagnosis of overweight/obesity, and this was the main reason for the referral in 9%. In total, 84% of the patients perceived obesity as a disease, while 78% considered losing weight was their responsibility. A remarkable 70% of the participants had received no education on implementing lifestyle changes from primary care; 10% were on weight loss medications. The barriers identified for losing weight are the low introspection of the disease (26%) and having an inadequate diet (30%) and lack of information (30%).

**Conclusions**: Even though most of the respondents perceive obesity as a disease, a high percentage did not have healthy habits (such as a carbohydrate rich diet) and the majority of patients lacked education about weight loss. Obesity is an unrecognized problem in our community with only 9% of the patients referred for specific weight assessment.

### 2.3. Correlation of Hyperuricemia with Left Ventricular Ejection Fraction, Risk Factors for and Documented Coronary Artery Disease in Patients Admitted with Acute Heart Failure

LesoJohn^1^ItaniWafic^1^HarutyunyanMisak^1^PatelHiren^2^TorosoffMikhail^2^1Department of Internal Medicine, Graduate Medical Education Office, Albany Medical College, 43 New Scotland Avenue, Albany, NY, USA2Department of Cardiology, Albany Medical College, 43 New Scotland Avenue, Albany, NY, USA

**Purpose**: Hyperuricemia has been associated with increased cardiovascular mortality and readmissions in patients with congestive heart failure (CHF) and increased incidence of coronary artery disease (CAD) in the general population. However, it is unclear whether hyperuricemia is a cause or a marker for CAD and CHF risk factors such as hypertension, dyslipidemia, and diabetes. 

**Method**: Hyperuricemia has been associated with increased cardiovascular mortality and readmissions in patients with congestive heart failure (HF) and increased incidence of coronary artery disease (CAD) in the general population. However, it is unclear whether hyperuricemia is a cause or a marker for CAD and CHF risk factors such as hypertension, dyslipidemia, and diabetes.

**Results:** Of 586 patients, 39% (226) were females, 68 (+/−15) years old on average, 40% (236) with history of diabetes, 70% (410) with treated hypertension, 47% (275) with history of coronary artery disease, and 37% (218) with history of chronic kidney disease (CKD), including those on hemodialysis. Hyperuricemia was not associated with increased prevalence of diabetes, hyperlipidemia, hypertension, or prior known CKD. However, prevalence of decreased GFR <60 mL/min was significantly higher in hyperuricemia (UAQ1 46%, UAQ2 66%, UAQ3 79%, and UAQ4 74%, *p* = 0.011). Prevalence of CAD documented by history, stress testing, or CT scanning was not affected by hyperuricemia (UAQ1 58%, UAQ2 54%, UAQ3 57%, and UAQ4 60%, *p* = 0.848), but there was increased prevalence of decreased <40% LV ejection fraction in CHF patients with hyperuricemia (UAQ1 29%, UAQ2 46%, UAQ3 48%, and UAQ4 54%, *p* = 0.039).

**Conclusions**: In patients admitted with acute heart failure, hyperuricemia is associated with reduced LV ejection fraction and decreased renal function, independently of traditional CAD risk factors. Further studies of this subject and whether this association can be modified by uric acid lowering medications are needed.

### 2.4. Factors Associated with Disparities in Appropriate Statin Therapy in an Outpatient Inner City Population

RashidSana^1^Suero-AbreuGiselle A.^1^KaratasakisAris^1^,^2^TysarowskiMaciej^1^DouglasAnalise Y.^1^,^3^PatelRicha^4^SiddiquiEmaad^5^BhardwajAishwarya^6^GerulaChristine M.^6^MatassaDaniel^1^1Department of Medicine, Rutgers New Jersey Medical School, Newark, NJ 07103, USA2Department of Medicine, Division of Cardiology, University of Washington, Seattle, WA 98195, USA3Division of Cardiology, Department of Medicine, University of Connecticut, Hartford Hospital, Hartford, CT 06102, USA4Department of Medicine, Icahn School of Medicine at Mount Sinai, New York, NY 10029, USA5Department of Medicine, NYU Langone Medical Center, New York, NY 10016, USA6Division of Cardiology, Department of Medicine, Rutgers New Jersey Medical School, Newark, NJ 07103, USA

**Purpose:** Appropriate lipid-lowering therapies are essential for the primary and secondary prevention of atherosclerotic cardiovascular disease (ASCVD). The aim of this study is to identify discrepancies between cholesterol management guidelines and current practice in an underserved population, with a focus on statin treatment.

**Method:** We reviewed the records of 1042 consecutive patients seen between August 2018 and August 2019 in an outpatient academic primary care clinic. Eligibility for statin and other lipid-lowering therapies was determined based on the 2018 American Heart Association and American College of Cardiology multisociety guideline on the management of blood cholesterol.

**Results:** Among 464 statin-eligible patients, age was 61.1 Â ± 10.4 years and 53.9% were female. Most patients were Black (47.2%), followed by Hispanic (45.7%), and White (5.0%). Overall, 82.1% of patients were prescribed a statin. Statin-eligible patients who qualified based only on a 10-year ASCVD risk >7.5% were significantly less likely to be prescribed a statin (32.8%, *p* < 0.001). After adjustment for gender and health insurance status, appropriate statin treatment was independently associated with age > 55 years (OR = 4.59 (95% CI 1.09–16.66), *p* = 0.026), hypertension (OR = 2.38 (95% CI 1.29–4.38), *p* = 0.005) and chronic kidney disease (OR = 3.95 (95% CI 1.42–14.30), *p* = 0.017). Factors independently associated with statin undertreatment were Black race (OR = 0.42 (95% CI 0.23–0.77), *p* = 0.005), and statin-eligibility based solely on an elevated 10-year ASCVD risk (OR = 0.14 (95% CI 0.07–0.25), *p* < 0.001). Hispanic patients were more likely to be on appropriate statin therapy when compared to Black patients (86.8% vs. 77.2%). Only 26.2% of patients on statins had follow-up cholesterol levels for monitoring treatment efficacy.

**Conclusions:** Statin underprescription was seen in approximately one out of five eligible patients, and was independently associated with Black race, younger age, fewer comorbidities, and eligibility via 10-year ASCVD risk only. Hispanic patients were more likely to be on appropriate statin therapy compared to Black patients.

### 2.5. Patiromer to Enable Spironolactone in Patients with Resistant Hypertension and Chronic Kidney Disease (AMBER): Results in the Prespecified Subgroup with Diabetes

AgarwalRajiv^1^RossignolPatrick^2^ArthurSusan^3^ConradAnsgar^3^WhiteWilliam B.^4^WilliamsBryan^5^1Indiana University School of Medicine, Indianapolis, IN, USA2University of Lorraine and FCRIN INI-CRCT, Nancy, France3Relypsa, Inc., a Vifor Pharma Group Company, Redwood City, CA, USA4University of Connecticut School of Medicine, Farmington, CT, USA5University College London (UCL), London, UK

**Purpose**: Spironolactone (SPIRO) reduces BP in patients with resistant hypertension (RHTN); however, its use in patients with advanced chronic kidney disease (CKD) is often limited by hyperkalemia. In AMBER, patiromer enabled more persistent use of SPIRO in patients with RHTN and CKD. As SPIRO is recommended in RHTN, and diabetes mellitus (DM) increases hyperkalemia risk, we report results in prespecified subgroups with Type 1 or 2 DM and without DM.

**Method**: AMBER was a randomized, double-blind, placebo (PBO)-controlled trial in adults with RHTN and eGFR 25 to ≤45 mL/min/1.73m². Patients were assigned (1:1) to PBO or patiromer at a starting dose of 8.4 g once daily, and SPIRO 25 mg once daily. Dose titrations were permitted after 1 week for patiromer/PBO to address hyperkalemia or hypokalemia: upward adjustment to 16.8 g QD, and then 25.2 g QD for local laboratory serum K+ >5.1 mEq/L, and downward adjustment for serum K+ <4.0 mEq/L. Dose of SPIRO was increased to 50 mg QD at week 3 in patients with serum K+ ≤5.1 mEq/L if systolic AOBP remained ≥120 mmHg. The primary endpoint (between-group difference at week 12 in the percent of patients on SPIRO) was assessed prospectively in prespecified subgroups by DM status.

**Results**: Overall, 295 patients were randomized, 145 (49%) with DM and 150 (51%) without DM. Baseline mean (SD) serum K+ (mEq/L) was 4.76 (0.34) in patients with DM and 4.67 (0.39) in patients without DM. Significantly more patients treated with patiromer than with PBO remained on SPIRO at week 12. In the subgroup with DM, 83.6% of patients receiving patiromer remained on SPIRO at week 12 compared with 65.3% of patients receiving PBO (between-group absolute difference = 18.3%, 95% confidence interval (CI) 4.4−32.2; *p* = 0.0111). In the subgroup without DM, 87.8% of patients receiving patiromer remained on SPIRO at week 12, compared with 67.1% of patients receiving PBO (between-group absolute difference = 20.7%, 95% CI 7.8−33.7; *p* = 0.0024). The least squares mean (SE) cumulative SPIRO dose in both subgroups was higher with patiromer than PBO, by 438.7 (177.7) mg in the subgroup with DM and 317.8 (175.0) mg in the subgroup without DM. Adverse events occurred in 61% (PBO) and 60% (patiromer) of patients with DM and in 46% (PBO) and 51% (patiromer) of patients without DM. Four patients had serum magnesium <1.4 mg/dL between baseline and week 12 (none < 1.2 mg/dL), including three with DM (1 PBO, 2 patiromer) and one without DM (patiromer) patients. In two of these patients with DM, serum magnesium was below the lower limit of normal (LLN; 1.8 mg/dL) at baseline. None of these patients had cardiac arrhythmias temporally associated with low magnesium levels, neuromuscular abnormalities, or serum K+ below the LLN (3.5 mEq/L).

**Conclusions**: Patiromer enabled more patients with advanced CKD and RHTN to continue treatment with SPIRO, regardless of DM status.

### 2.6. Hospital and Emergency Department Utilization in US Veterans with Hyperkalemia

KovesdyCsaba P.^1^GosmanovaElvira O.^2^WoodsSteven D.^3^FogliJeanene J.^3^RowanChristopher G.^4^HansenJared L.^5^SauerBrian C.^5^1University of Tennessee, Memphis, TN, USA2Albany Medical College, Albany, NY, USA3Relypsa, Inc., a Vifor Pharma Group Company, Redwood City, CA, USA4COHRDATA, Santa Monica, CA, USA5Salt Lake City VA Medical Center (IDEAS), Salt Lake City, UT, USA

**Purpose:** Hyperkalemia (HK) is a potentially life-threatening metabolic disorder and a challenging clinical problem for clinicians caring for patients with chronic kidney disease (CKD), diabetes mellitus (DM) and heart failure (HF). HK is common in older patients with cardiorenal comorbidities and often limits the use of guideline recommended Renin Angiotensin Aldosterone System inhibitors (RAASi) medications. Patiromer is a sodium-free, non-absorbed potassium (K+) binder (KB) approved for the treatment of hyperkalemia (HK). Here, we aim to describe electrolyte-related Healthcare Resource Utilization (HRU) in Veterans with HK who initiated patiromer or discontinued RAAS inhibitor (RAASi DC) therapy and were not receiving a KB (1/1/2016−8/30/2018).

**Method**: Using retrospective, observational data, patients utilizing a Veterans Affairs hospital or Emergency Department (ED) during the 6 months prior to the index date were assessed at 1, 3, and 6 months post-index. The index date was the date of patiromer initiation or the date of RAASi DC in patients not receiving a KB (RAASi DC/no KB). All patients had a baseline serum K+ ≥5.1 mEq/L and HF, DM, or non-dialysis dependent CKD. Follow-up began at index date and ended at first censoring event (discontinuation or switch of index KB, death, end of follow-up, or 6 months post-index). Patients with continuous exposure to patiromer and those who did not restart RAASi were analyzed.

**Results:** In total, 288 and 26,543 patients were included in the patiromer and RAASi DC/no KB groups, respectively. At baseline, the mean age was 70 years (patiromer) and 72 years (RAASi DC/no KB) with the majority of patients being male (98%) and 24% and 15% African-American (patiromer and RAASi DC/no KB groups, respectively). In both cohorts, 83% of patients had DM at baseline, while a higher percentage of patients had advanced CKD, HF and greater utilization of the ED and hospital in the patiromer group. When evaluating patients with an electrolyte-related hospitalization or ED visit in the pre-index period, we observed no electrolyte-related hospitalizations or ED visits in patients with continuous exposure to patiromer at 1, 3, and 6 months post-index. In the RAASi DC/no KB group, 2–5% of patients reutilized the ED or hospital within 6 months.

**Conclusions**: At baseline, more than 80% of both cohorts had a diagnosis of diabetes but in the patiromer cohort a greater percentage of patients had heart failure, advanced CKD, and a higher utilization of the ED and hospital at baseline. We observed that patients continuously exposed to patiromer at 1, 3, and 6 months did not experience any additional utilization of the ED or hospital after initiation of treatment. This is a descriptive observational study so no comparative or causal claims can be made and given the limited number of patiromer users, additional investigation is warranted.

### 2.7. Type-2 Diabetes Medication Utilization—A Regional Health Plan’s Analysis of Utilization Since the American Diabetes Association’s Treatment Guidelines Update in 2019

LorsonMarkButterfieldKristenSepeKaylaMahadevanGokulakrishnanBansalSudhirNeighborhood Health Plan of Rhode Island and Endocrinologist, Kent Hospital, Smithfield, RI, USA

**Purpose**: The American Diabetes Association (ADA) recently updated their recommendations on treatment guidelines for type-2 diabetes (T2DM). As is the case with most changes to treatment guidelines, it takes time for providers to shift their prescribing patterns. Neighborhood Health Plan of Rhode Island (Neighborhood) is a regional health plan that provides Medicaid, Commercial (through the state’s Health Source RI Exchange), and Medicare-Medicaid (MMP or dual eligible) coverage for Rhode Islanders. In 2019, Neighborhood allowed for greater access on its Medicaid, Commercial, and MMP formularies for sodium-glucose cotransporter-2 inhibitors (SGLT2) and glucagon-like peptide-1 receptor agonist (GLP-1) medications. Since making the formulary updates, Neighborhood wanted to determine how providers and members had responded to the updated treatment guidelines. To achieve this objective, Neighborhood reviewed claims data to determine if SGLT2s or GLP-1s were added to medication regimens. Additionally, Neighborhood wanted to gain knowledge of its provider network’s T2DM prescribing patterns in relation to a member’s age, chronic kidney disease (CKD) status, or atherosclerotic cardiovascular disease (ASCVD) status. Securing and understanding these results will help Neighborhood determine how best to provide targeted provider education moving forward and, in turn, strengthen Neighborhood’s ability to positively impact its member population.

**Method**: Neighborhood analyzed claims data from January 2019 through June 2020 for members with T2DM continuously enrolled in the plan since July 2018. Medication utilization was pulled based on Generic Product Identifier (GPI). Drug Category was based on the medication’s mechanism of action. The diagnoses of CKD (N18-N18.7) and ASCVD (G45, I20-I25, I25.10, I60-I66, I67.2, I67.81-I67.84, I69, I70, I73.9-I75, I99, R29.7, Z86.7, Z98.6, Z95.1, Z95.5, Z95.8, and Z95.9) were determined based on claims data submitted with ICD-10 codes during the same time period. Prescriber specialty was identified by how the prescriber was registered within the Pharmacy Benefit Manager’s claims system.

**Results**: The analysis showed that 80.5% of members with T2DM identified (n = 7663) were on metformin, 38.2% were on a sulfonylurea, 31.1% were on a long-acting insulin, 12.8% were on a rapid-acting insulin, 10.5% were on a dipeptidyl peptidase-4 inhibitor (DPP-4), 10.3% were on a GLP-1, and 5.6% were on an SGLT2. Of members with CKD (n = 1115), 51.6% were on metformin, 48.9% were on a long-acting insulin, 33.5% were on a sulfonylurea, 27.1% were on a rapid-acting insulin, 13.2% were on a DPP-4, 11.4% were on a GLP-1, and 5.5% were on an SGLT2. Of members with ASCVD (n = 2625), 71.8% were on metformin, 39.1% were on a long-acting insulin, 36.6% were on a sulfonylurea, 17.5% were on a rapid-acting insulin, 12.6% were on a DPP-4, 10.9% were on a GLP-1, and 6.4% were on an SGLT2. Of members 65 years of age and older (n = 1912), 70.4% were on metformin, 37.3% were on a sulfonylurea, 31.5% were on a long-acting insulin, 17% were on a DPP-4, 14.6% were on a rapid-acting insulin, 6% were on a GLP-1, and 4.9% were on an SGLT2. Of members 40 to 64 years of age (n = 5142), 83.7% were on metformin, 39.5% were on a sulfonylurea, 30.7% were on a long-acting insulin, 11.8% were on a rapid-acting insulin, 11.4% were on a GLP-1, 8.6% were on a DPP-4, and 6% were on an SGLT2. Of members under 40 years of age (n = 609), 85.1% were on metformin, 33.7% were on a long-acting insulin, 30.5% were on a sulfonylurea, 15.6% were on a rapid-acting insulin, 14.1% were on a GLP-1, 6.7% were on a DPP-4, and 4.9% were on an SGLT2. 

**Conclusions**: For the identified members with T2DM, metformin is the most utilized anti-diabetic medication, followed by sulfonylureas and long-acting insulins. Although this may have been a previous ADA-recommended treatment strategy, it is no longer current. In regard to members with CKD, although it makes sense that only 51.6% of the patients are taking metformin (potentially due to low creatinine clearance levels), further analysis will need to occur for the 33.5% of patients taking a sulfonylurea to ensure appropriate utilization. Additionally, younger patients (under 40 years old) are more likely to be on a GLP-1 (14.1% vs. 11.4% and 6%) and less likely to be on a sulfonylurea (30.5% vs. 39.5% and 37.3%) or a DPP-4 (6.7% vs. 8.6% and 17%) compared to members 40 to 64 or over 65 years old, respectively. Within these age group comparisons, each age group was similar in their utilization of long-acting insulin (33.7% vs. 30.7% vs. 31.5%) and rapid-acting insulin (15.6% vs. 11.8% vs. 14.6%). This analysis helps to understand that changes in guidelines do not immediately lead to changes in utilization and provides a blueprint for Neighborhood to help ensure appropriate T2DM treatment.

### 2.8. Adherence to American Diabetes Association Guidelines in a Student-Run Free Clinic for Migrant Farmworkers

GulaniAaishwariyaMominAamirEl-SaidAngieTapasakBrandonGuptaRemaCollege of Medicine, University of Central Florida, Orlando, FL, USA

**Purpose**: Chronic conditions such as diabetes can prove to be burdens for patients with little to no access to constant healthcare. Such conditions must be monitored to prevent further harm to the patients’ wellbeing. Clinics such as the Apopka Farmworkers Clinic provide free care at the Farmworkers’ Association of Florida in Apopka four times a year and now biweekly as telehealth. At the clinic, patients visit to receive routine work ups with the internal medicine team, vaccines, and specialized care. Diabetes has been found to be one of the top four most seen conditions at the clinic, the others being obesity, hypertension, and high cholesterol. Such conditions are chronic and should be monitored to prevent adverse outcomes with routine and adequate healthcare, most of which these patients do not receive elsewhere. Thus, by providing care, education, and prescribing lifesaving drugs, the clinic is able to aid these patients with their chronic conditions and prevent further complications. This project looks at how the clinic provides the standard of care (SOC) as dictated by the American Diabetes Association (ADA) for diabetic patients. It aims to reveal adherence to guidelines for these underserved patients, and determine if the free clinic provides them with the same quality of care as our hospital counterparts.

**Method**: The objective of this project is to see if the Apopka Farmworkers Clinic follows our SOC as stated by the ADA. The study conducted will be an analysis of patients presenting with diabetes at the Apopka Farmworkers Clinic from 2018 to 2020. Visits studied will include all clinic visits and telehealth visits conducted during this time period. Measurements at their visits will be retrospectively reviewed to determine the tests that were ordered and noted in charts. Adherence to SOC will be determined based on five criteria set out by the ADA: metabolic control, cardiovascular assessment, complications, lifestyle, and immunizations. Metabolic control covers HbA1c and other glucose measurements, if taken. Cardiovascular assessment includes blood pressure and lipid profile, including LDL, HDL, and triglycerides. Complications include retinopathy, and nephropathy, and others conditions, which should be checked or referred to our specialty clinics when available. The lifestyle component includes exercise monitoring and reporting. Lastly, immunizations determine if the influenza and pneumococcal vaccines have been administered. The variables studied will fall under these categories and include HbA1c, lipid panel tests, blood pressure, demographical information, family histories, immunizations, and specialist evaluations from the clinic visits. A score will be given for each patient’s care plan during their visit with one point for each of the five categories listed. 

**Results**: The retrospective chart review of 24 diabetic patients revealed an average age of 54.38 (Â ± 13.65) years. There were 7 males and 17 female patients observed. The race breakdown was 20.83% Caucasian, 25.00% African American, 20.83% Hispanic, and 29.16% unreported. Further analysis will be conducted to determine patients whose care adhered to all five categories of diabetic monitoring, 4 of the 5 categories, and so on. The category most fulfilled for patient visits will also be noted to determine which area the clinic consistently provides care in, as dictated by the ADA’s SOC.

**Conclusions**: These results will aid in aligning our clinic’s procedures with the ADA’s SOC for patients with diabetes to ensure clinic patients are receiving the same level of care as those at other locations. Results will also show the degree of uncontrolled diabetics present in our free clinic versus the national average based on lab results. We hope to improve the care of diabetic patients entering our clinic through this project by analyzing the current standard of care and comparing ours to the standards set by the ADA. This knowledge will provide information to future clinics and physicians who serve this patient population.

### 2.9. Cardiovascular Risk Factor in the Staff of Public Hospital 

LuzDon GraciaLorenaCabrol ObaidNataliaFernádezServicio de Cardiología, Hospital San Martín, Paraná, Entre Ríos, Argentina

**Purpose**: In regard to morbidity and mortality, cardiovascular diseases are the most common causes worldwide. They form part of non-communicable chronic diseases (NCDs) and in Argentina they represent the most prevalent cause of death (39.3%). Smoking, inadequate feeding, physical inactivity and the harmful use of alcohol are the result of personal and social behavior acquired and influenced by the environment, the availability and promotion of harmful products (tobacco, alcohol, unhealthy food and drinks, with high levels of sugar, fat and salt). These toxic habits such as smoking, or cultural habits such as sedentary lifestyle and other illnesses such as hypertension, dyslipidemia, diabetes, overweight and obesity constitute the risk factors developing cardio-vascular diseases. The object of this study was to assess the current prevalence of cardiovascular risk factors within the staff of the Hospital San Martin of Parana, to carry out a comparison with the previous study in 2014 as well as with the fourth national survey of risk factors (2018) to monitor the evolution of the main risk factors.

**Method**: In total, 400 people were studied through anonymous self-administered surveys. The data required were essential to determine age, sex, size, weight, arterial hypertension or hypotensive treatment, overweight or obesity, sedentary lifestyle, diabetes, dyslipidemia or smoking. Smokers were also asked to complete the Modified Fagestrom Test to value the degree of nicotine dependence. The surveys were answered by health professionals, administrators, nursing staff and general services. The Stata statistical software was implemented for the descriptive analysis of the sample and to prove the hypothesis of difference between risk factors in a previous evaluation and in the national survey. There, the work was carried out with a test of independence and with differences within proportions.

**Results**: Proportionally, 65% of this sample corresponds to women and 35% to men; 53% of this sample is within the 20–40-year-old range; 3% is 61 years old or over; and the rest (44%) are between 41 and 60 years old. Prevalence of arterial hypertension represented 17%, while overweight and obesity accounted for 51%. Sedentary lifestyle was observed in 52% of the surveyed people. Diabetes prevalence was 7% while 15% presented dyslipidemia antecedents. Smoking prevalence corresponded to 27%, 63% denoted low dependence on nicotine and only 7% of the same group presented high dependence. In regard to the relation between risk and sex factors, there was a greater prevalence of obesity and smoking in men. The group aged 61 and over presented a prevalence in all risk factors except in obesity at which there is a higher prevalence in the 41–50-year-old group. In respect of the younger ones (20–40 years old) the most notable risk factors were sedentary lifestyle and smoking. Comparing the evaluation carried out in 2013 over a sample of 448 people of similar distribution regarding sex and age, sedentary lifestyle was the most different factor corresponding to 35% in 2013, against 52% at present. A comparison of these results with those of the national survey concluded that sedentary lifestyle and overweight /obesity were the most prevalent; though in a smaller percentage, a higher percentage of smokers than in the National survey (28% vs. 22.2%) was observed, as was a lesser prevalence of hypertension, dyslipidemia, and diabetes in hospital population.

**Conclusions**: The risk factors mentioned before were compared in regards to the previous study carried out in 2014, all with a 5% level of significance and it was statistically proved that there had been an increase in sedentary lifestyle which went from 35% to 52% (value *p* = 0.000001). The remaining risk factors showed insignificant statistical differences, thus, no variation existed. The p values obtained were: obesity (*p* = 0.21), hypertension (*p* = 0.71), diabetes (*p* = 0.37) smoking (*p* = 0.20) and dyslipidemia (*p* = 0.17). Concerning the last sample considered, a test of independence was done to determine if the risk factors depended on sex and age. The conclusion was that there exists independence with a value *p* = 0.3362 for sex and dependence for age (*p* < 0.001). When carrying out a test of the hypothesis of proportions, equality of prevalence was demonstrated in hypertension, sedentary lifestyle, diabetes and dyslipidemia with p values of 0.88, 0.76, 1.00 and 0.08, respectively, according to sex. Obesity and smoking are statistically different for sex. The differences between the average of national surveys and those carried out at Hospital San Martín (HSM) were statistically proved in terms of hypertension, dyslipidemia, overweight /obesity and diabetes factors. The first two factors showed values inferior to 0.0000001, while overweight/obesity and diabetes had p values of 0.003. The other two factors (sedentary lifestyle and smoking) cannot prove to be different from the national media attained in the surveys. Upon the analysis of the results, it can be stated that the factor prevalence favoring cardiovascular diseases has kept similar levels with time while sedentary lifestyle and overweight /obesity have augmented. 

### 2.10. Sex and Age-Based Differences in Fasting HOMA-IR among Patients with Mild to Moderate Myalgic Encephalomyelitis/Chronic Fatigue Syndrome: Novel Approach to Glucose/Insulin Anomalies in ME/CFS

AssilRahaf Al^1^LewisDonald^2^McGregorNeil^2^1University of British Columbia, Vancouver, BC, Canada2University of Melbourne, Melbourne, Victoria, Australia

**Purpose**: Glucose and insulin anomalies have been identified in subjects with defined Myalgic Encephalomyelitis/Chronic Fatigue Syndrome (ME/CFS). A few studies have assessed the plasma glucose and insulin levels and found a higher likelihood of insulin-induced hypoglycemia and insulin resistance in ME/CFS patients compared to healthy controls. We examined the differences in plasma glucose and insulin trends among sex and age groups, the incidence rate of insulin-induced hypoglycemia, and the association between sex and age and insulin resistance in patients with mild to moderate ME/CFS.

**Method**: We performed a retrospective analysis of data from CFS Discovery Clinic Melbourne, Australia. The independent variables of interest were age and sex. The primary outcomes were (1) repeated measures of plasma glucose and insulin at 0, 30, 60 min of the Homeostatic Model Assessment of Insulin Resistance (HOMA-IR), and (2) insulin resistance defined as fasting HOMA-IR > 2. The incidence rate of insulin-induced hypoglycemia was reported. After adjusting for Body Mass Index (BMI), we reported the standard errors (SEM) and p values of plasma glucose and insulin trends using a multivariable mixed-effect linear regression model and odds ratios for insulin resistance using a multivariable logistic regression model.

**Results**: We included 1108 patients in our analysis; 72% were females, 10% were young (less than 18 years old), 44% had BMI ≥ 25, and 35% had insulin resistance. At 60 min, 155 (20%) of 775 patients had insulin-induced hypoglycemic response (mean = 4 mmol/L) compared to their fasting glucose (mean = 4.6 mmol/L). Of those, 75% were females. In the adjusted mixed-effect linear regression, plasma glucose trends were (β, 0.36; SEM, 0.1; *p* = 0.0003) higher in males compared to females, whereas plasma insulin trends differences were non-significant. Plasma insulin trends were (β, −0.14, SEM, 0.03; *p* < 0.0001) lower in >18-year-old patients compared to those < 18 years old, whereas plasma glucose trend differences were non-significant. Young age (adjusted OR 4.42; 95% CI 2.40 to 8.14) and males (adjusted OR 1.27; 95% 1.03 to 1.48) were both associated with higher odds of insulin resistance. 

**Conclusions**: In patients with mild to moderate ME/CFS, males and patients who are < 18 years old had higher odds of insulin resistance than others. Females predominantly developed insulin-induced hypoglycemic response at 60 min. Sex- and age-based differences in glucose/insulin anomalies may exist in ME/CFS patients and may be a target for genetic susceptibility and personalized diagnosis and management in ME/CFS patients. 

### 2.11. Impact of a Multidisciplinary Diabetes Care Team in Primary Care Settings on Glycemic Control

HandlowNicole^1^NoltonBrittany^1^WinterSarah^1^WesselCynthia^1^PennockJennifer^2^1Allegheny General Hospital Department of Pharmacy Services, Pittsburgh, PA, USA2Allegheny Health Network, Pittsburgh, PA, USA

**Purpose:** A multidisciplinary team approach to diabetes management involves various healthcare members to optimize patient outcomes. However, there are limited studies that look at the effect of a multidisciplinary team on diabetes outcomes outside of endocrinology services. This study aimed to evaluate the impact of a primary care multidisciplinary team on glycemic control and additional risk reduction during a 12-week diabetes program.

**Method:** Retrospective review of medical records for patients who completed the 12-week diabetes program at three primary care clinics from January to August of 2018 occurred. The primary outcome was mean change in hemoglobin A1c (HbA1c). Secondary outcomes included 24-week HbA1c, mean change in blood pressure, weight, Diabetes Distress Scale (DDS) score, and Patient Health Questionnaire (PHQ9) score. Other secondary objectives included the number of patients on statin therapy and ACE inhibitor or angiotensin receptor blocker therapy as indicated. Patients enrolled in the diabetes program were seen by a provider, pharmacist, dietician, and behavioral health specialist at an initial visit and a 12-week follow-up visit. Follow-up occurred either telephonically or face-to-face in the interim for lifestyle counseling and medication adjustments. 

**Results:** In total, 85 patients were included in the final analysis. At 3-month follow-up, the mean A1c was significantly decreased (2.1 +/− 2.3, *p* < 0.001), 54.1% of patients met their A1c goal (*p* < 0.001), 80.5% were on statin therapy (*p* < 0.001), and the mean change in weight (kg) was −1.96 +/− 3.9 (*p* = 0.001) from baseline.

**Conclusions:** This study demonstrates that a multidisciplinary approach to diabetes management can reduce HbA1c and other risk factors. A multidisciplinary model can be replicated in primary care offices to allow for comprehensive care and improvement in health outcomes for a broader patient population who may not have access to specialty services.

### 2.12. Review of Noninvasive Testing for Hepatic Steatosis as a Marker for Metabolic Disease

ShawSandyAnselmoMichaelAmadorSteveLufkinRobertDepartment of Radiology, USC Keck School of Medicine, Los Angeles, CA, USA

**Purpose**: Hepatic steatosis is a primary marker of metabolic disease often predating other symptoms. It is estimated to affect a large proportion of the adult US population. We review strategies and challenges for noninvasive detection of hepatic steatosis. 

**Methods:** Examples of noninvasive techniques for the detection and quantification of hepatic steatosis including blood tests, ultrasound, computed tomography (CT) and magnetic resonance imaging (MRI) and spectroscopy (MRS) were reviewed. The advantages and disadvantages of each approach were reviewed.

**Results**: Liver enzymes are relatively nonspecific in many patients with simple steatosis. Conventional ultrasound has the advantage of no ionizing radiation but is operator dependent with limited quantification of steatosis. New systems offer more features and the possibility of detecting steatohepatitis. CT has the disadvantage of ionizing radiation; however, quantification is possible and semiautomated artificial intelligence powered systems can now be applied retrospectively to large databases of CT Abdomen studies. MR systems have no ionizing radiation and can perform steatosis quantification with multi-echo gradient echo sequences. Single purpose MRI hepatic steatosis examinations could potentially be done rapidly at relatively low cost. MR spectroscopy can perform quantification, but limited availability and high cost limit its application. 

**Conclusions:** Blood tests for liver enzymes are relatively nonspecific for simple steatosis. Ultrasound, CT, and MR all offer possibilities for hepatic steatosis detection with various advantages and disadvantages depending on the situation. 

For more information, please visit: https://www.cardiometabolichealth.org/

